# Mixed Plate Ductal Intrahepatic Cholangiocarcinoma Mimicking Hepatocellular Carcinoma

**DOI:** 10.7759/cureus.52992

**Published:** 2024-01-26

**Authors:** Sania Ajmal, Srujan Edupuganti, Adiraj Singh

**Affiliations:** 1 Internal Medicine - Pediatrics, Hurley Medical Center - Michigan State University, Flint, USA

**Keywords:** intrahepatic cholangiocarcinoma, diagnostic testing, portal vein thrombosis and malignancy, hepatitis c (hcv) infection, ductal plate malformation

## Abstract

Cholangiocarcinoma (CCA) refers to malignancies of the bile ducts that arise in the intrahepatic, perihilar, or distal (extrahepatic) biliary tree, excluding the gallbladder and ampulla of Vater. Although rare, the majority of these cancers are locally advanced at presentation, making them extremely fatal. We present a case of a 65-year-old man who came in for abdominal pain and ascites and was found to have portal vein thrombosis. Initial imaging showed a hepatic lesion, raising suspicion of a hepatic malignancy. Despite the multiple imaging modalities used, the diagnosis remained uncertain. Eventually, a biopsy of the lesion showed it to be a variant of intrahepatic CCA, which has mixed features of hepatocellular carcinoma and CCA. This variant is highly malignant and poorly responsive to treatment, leading to a poor prognosis. Our patient on diagnosis was categorized as stage 4 cancer, and although treatment was initiated, she succumbed to the disease in six months. Based on our experience with this patient, we would like to highlight the significance of a multimodal imaging approach and the necessity of tissue diagnosis when the initial work-up is inconclusive since these factors will also impact the course of management.

## Introduction

Biliary tract cancers (BTCs) comprise <1% of all cancers but constitute 10-15% of primary liver cancers and are usually diagnosed late with a poor prognosis [1-4]. The average survival rate in a five-year period for intrahepatic BTC is 24% for early-detected tumors vs. 9% for cancers with lymphatic metastasis. They can be further distinguished by their tissue origin and geographical location. Combined cholangiocarcinoma (CCA) and hepatocellular carcinoma (HCC) with ductal plate malformation is a rare entity with unclear etiopathogenesis [5], which is a subtype of intrahepatic CCA. Distinction between these subtypes is necessary for appropriate management and prognostic evaluation, often requiring a biopsy as imaging alone can be insufficient for diagnosis. While the genetic characteristics are uncertain, ductal plate malformation remains an aggressive and treatment-resistant variant.

## Case presentation

**Figure 1:**
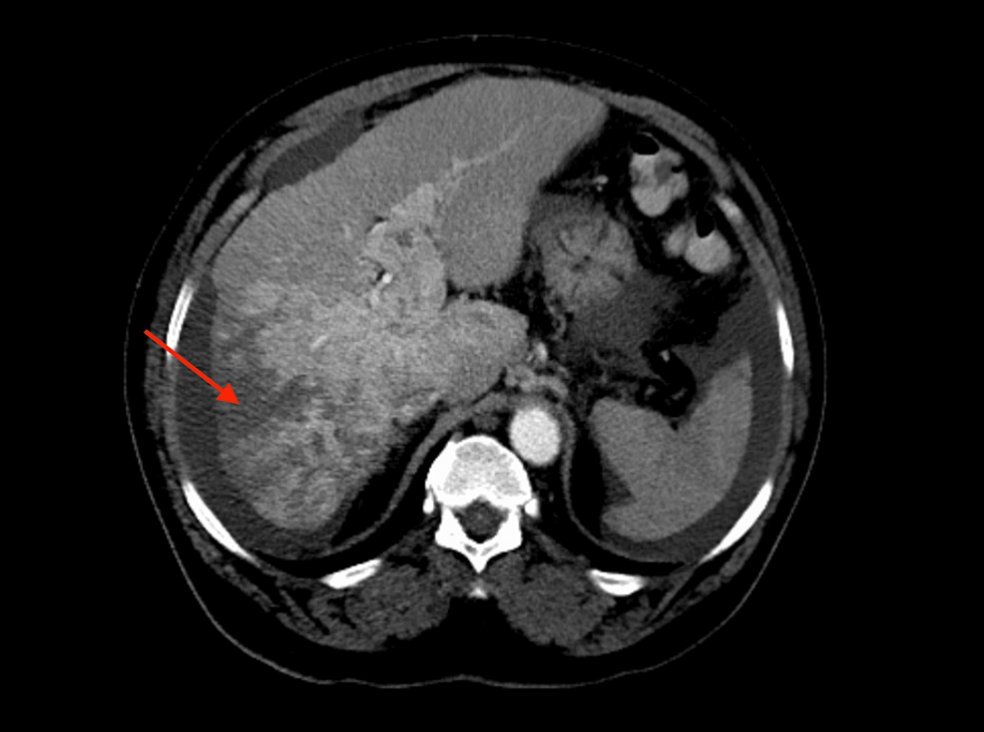
Transverse plane computed tomography (CT) image of the abdomen showing heterogenous liver lesion

**Figure 2:**
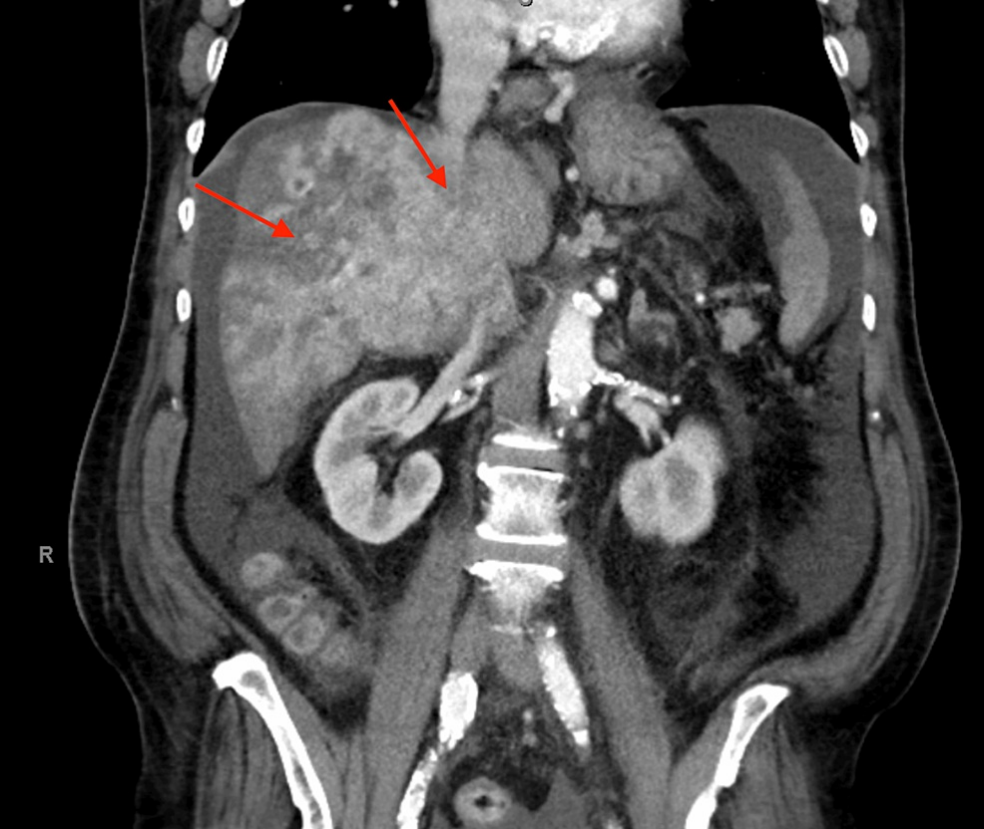
Coronal plane computed tomography (CT) image showing extensive liver metastasis

**Figure 3:**
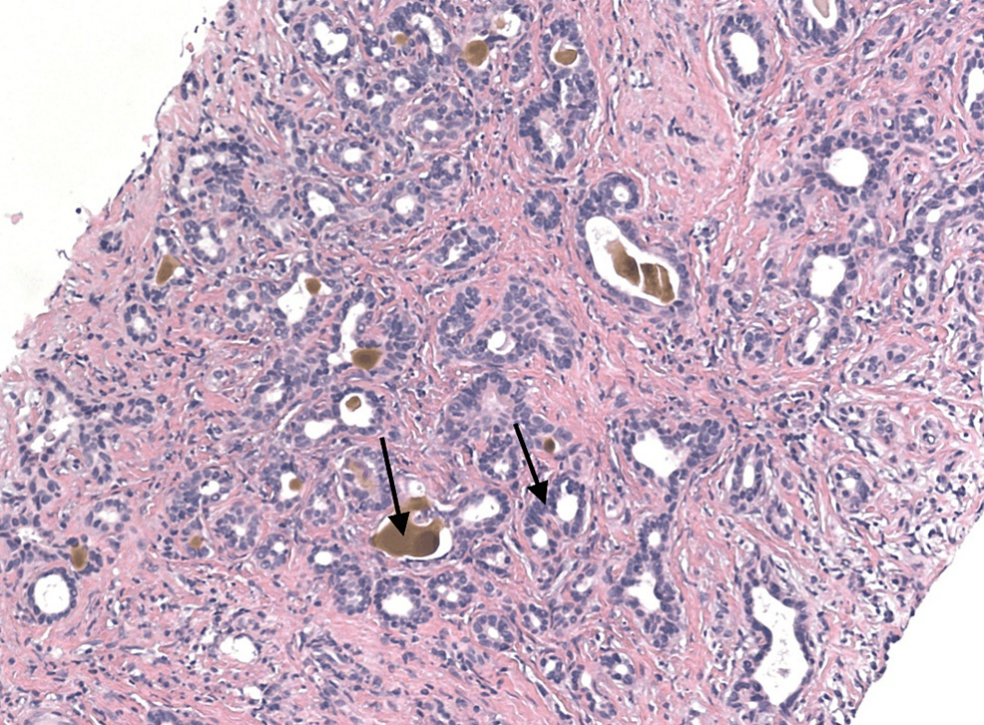
Histopathology showing neoplastic glands with nuclear atypia, entrapped bile (H&E x100)

**Figure 4:**
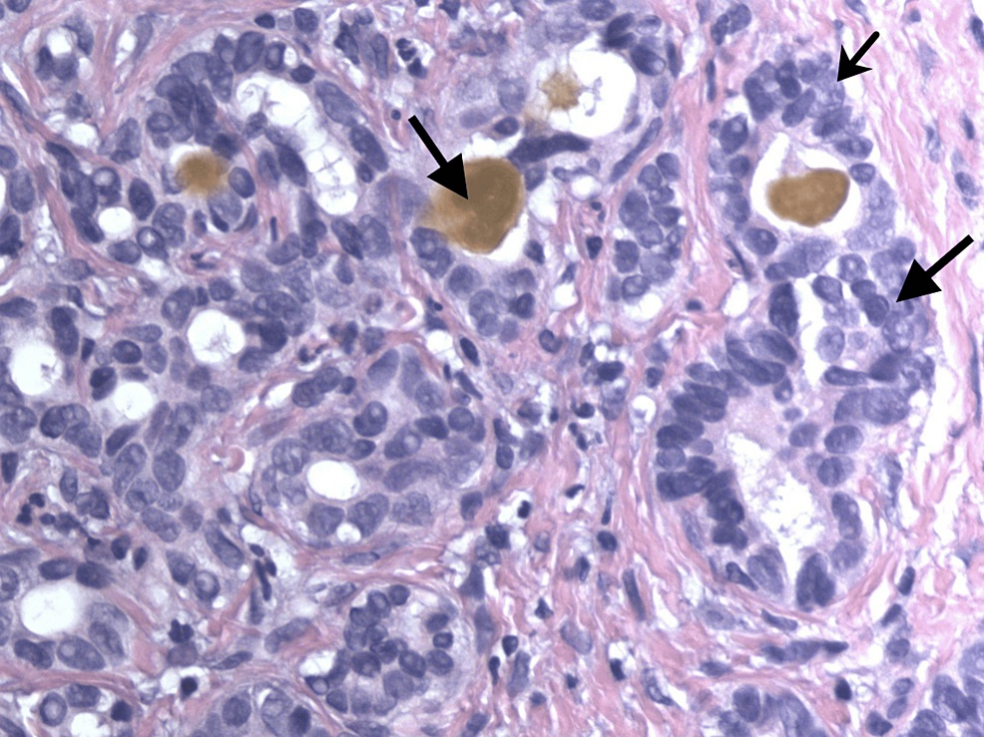
Histopathology showing entrapped bile and proliferation of glandular epithelial cells with nuclear atypia (H&E x400)

A 65-year-old African American male with a history of hepatitis C, treated over 10 years ago, presented to the emergency department with complaints of gradually progressive abdominal girth, right upper quadrant abdominal pain, and declining appetite and nausea for several weeks. The patient denied any alcohol or drug use. On examination, there was generalized abdominal distension and tenderness, along with fluid thrill and shifting dullness. He had no signs of chronic liver disease. Evaluation of the patient’s labs showed elevated alkaline phosphatase, aspartate aminotransferase, alanine aminotransferase, direct bilirubin, and normal total bilirubin. Prothrombin time and international normalized ratio were borderline prolonged (Table 1). Ultrasound of the abdomen was significant for mild hepatomegaly, ascites, extensive portal vein thrombosis, and gallbladder wall thickening. Paracentesis was performed to relieve the ascites, releasing 3.7 L of fluid. Diagnostics performed on the ascitic fluid showed a serum ascites albumin gradient greater than 1.1. Spontaneous bacterial peritonitis was ruled out, and empiric antibiotic coverage was discontinued. Single-phase CT imaging showed a heterogeneous right lobe of the liver suspicious of HCC, with another hyper-enhancing lesion seen in the left lobe of the liver measuring 1.4 cm (Figures 1-2). Portal venous thrombosis was seen, which was suspicious for a tumor thrombus. Blood work was sent for tumor markers, which revealed normal levels of alpha-fetoprotein (AFP) and elevated levels of cancer antigen 19-9 (CA 19-9) (Table 1). As per radiology recommendation, MRI was preferred to a multi-phase CT, which showed an under-distended gallbladder with surrounding ascites. Two areas of high T2 signal were noted in the liver, similar in region to the CT imaging, but no focal lesion could be characterized. Portal thrombosis extending bilaterally into the right and left branches, along with extensive right lobe involvement, was seen again. The MRI reported it to be a bland thrombus, and oral anticoagulant Lovenox, which had been started, was continued. A biopsy was recommended, which subsequently showed irregularly shaped glands within the dense fibrous stroma. Neoplastic glands showed nuclear atypia and entrapped bile, diagnostic for CCA. More specifically, the pathology testing was pathognomonic for the ductal plate malformation variant, which mimics HCC (Figures 3-4). The patient was diagnosed with stage 4 intrahepatic CCA and started on immunotherapy, later on in combination with chemotherapy. With minimal improvement and due to a worsening condition, the patient was switched to palliative chemotherapy. Subsequently, he succumbed to his disease and passed away.

**Table 1:** Lab results

Laboratory test	Results (reference values)
Alkaline phosphatase	160 IU/L (39-117)
Total bilirubin	0.8 mg/dl (6-24)
Direct bilirubin	0.4 mg/dl (<0.3)
Aspartate aminotransferase	64 IU/L (0-40)
Alanine aminotransferase	22 IU/L (0-44)
Serum albumin	3.8 g/dl (3.5-5.5)
Prothrombin time	13.0 seconds (10-13)
International normalized ratio	1.29 (0.9-1.15)
Partial thromboplastin time	25.1 seconds (25-36)
Ascitic fluid albumin	1.3 g/dl
Serum ascites albumin gradient	2.5
Alpha-fetoprotein	2.9 ng/ml (<9)
Cancer antigen 19-9	233 U/ml (<35)

## Discussion

BTCs are a rare form of cancer with an overall incidence of <1% of cancers, though they still bear a burden of 10-15 % of primary liver cancers [4]. BTCs can be classified as gallbladder carcinoma and CCA arising from the biliary tree. CCA can subsequently be divided anatomically into intrahepatic CCA arising from segmental bile ducts, perihilar CCA encompassing the perihilar bile ducts, including the right and left bile ducts, and distal CCA originating in the duct distal to the cystic duct. Among the intrahepatic CCA, combined features of HCC and CCA are seen in a now-recognized rare variant, the “ductal plate malformation variant,” which is prominent for its poor prognosis and aggressive nature [6].

BTCs are predominantly common in Asian countries and linked highly to fluke infestations and chronic inflammation, but currently, we are observing an increasing incidence of intrahepatic CCA in Western countries as well. This surge is being linked to increased chronic liver diseases and migration patterns. Common risk factors for intrahepatic CCA include hereditary conditions like primary sclerosing cholangitis, choledochal cysts, hepatobiliary flukes, hepatolithiasis, and hepatotropic viruses [7], especially hepatitis B and C. Some other factors, like diabetes, obesity, smoking, and alcohol [8,9] are also seen to be associated with the rising trend of malignancy, hinting at the underlying chronic inflammation and cirrhotic changes attributed to these carcinogenic transformations.

The presentation of patients for BTC is usually silent and late, with perihilar CCA and distal CCA being more symptomatic with jaundice, ascites, and other symptoms of regional obstruction. However, a patient with intrahepatic CCA is commonly diagnosed with an advanced-stage presentation like hepatic vein and portal vein thrombosis, as seen in our patient. Prognosis and survival are reportedly poor due to the late diagnosis and the advanced nature of the disease. Prognosis also depends on the underlying risk factors and alters the prognosis and survival post-treatment, as reported by a study showcasing the worse prognosis between patients having hepatitis B virus infection and those not having hepatitis B virus infection [7].

The dependence of prognosis and management options on early detection makes surveillance and correct diagnosis imperative. Current screening measures include six monthly ultrasounds of the liver in patients deemed at risk, like hepatitis B- and hepatitis C-positive patients. The initial diagnosis of the patient would commonly follow imaging by ultrasound, CT, or MRI with concerns for a lesion. For further assessment, current recommendations include imaging of cross sections of the chest, abdomen, and pelvis for evaluation of malignancy and metastatic status, with magnetic resonance cholangiopancreatography ideally able to assist in biliary tract visualization and analysis. Other modalities include endoscopic ultrasound or endoscopic retrograde cholangiopancreatography for closer inspection and evaluation and, in some cases, for collecting biopsy specimens [4].

Large intrahepatic CCA on imaging appears as a hypoattenuating mass with peripheral enhancement, capsular retraction, and biliary dilation, whereas small intrahepatic CCA is hyper-vascular, lacking the distinguishing peripheral enhancement in some cases mimicking HCC. Combined HCC and CCA add further difficulty to diagnosis by imaging as they have combined features and are often misdiagnosed, thus requiring a biopsy [10-12]. As in our patient, after ultrasound identified portal vein thrombosis in our patient, we began suspecting HCC. A CT scan was suspicious for HCC, but the results were inconclusive. Hence, an MRI was performed, which was indeterminate as well. Given the imaging results and normal AFP levels, we decided to go for a biopsy, which came back positive for CCA with ductal plate malformation.

## Conclusions

With the rising incidence of HCC and CCA, our index of suspicion needs to be higher, stressing the need for lifestyle changes and rigorous screening in high-risk patients for early diagnosis. Also, once a suspicious lesion is detected and one imaging modality is inconclusive, a multimodal imaging approach should be adopted, and a biopsy might be required as well if the results are still indeterminate. The prognosis and management are vastly different between HCC and CCA. Among CCAs for the ductal plate malformation variant, which is a rare but aggressive variant, this is even more important. Therapeutic decisions, including the agents that can be used and whether systemic therapy will be beneficial, are all dependent on molecular studies, thus requiring a thorough workup and diagnosis.
